# The Differential Role of Human Cationic Trypsinogen (*PRSS1*) p.R122H Mutation in Hereditary and Nonhereditary Chronic Pancreatitis: A Systematic Review and Meta-Analysis

**DOI:** 10.1155/2017/9505460

**Published:** 2017-10-08

**Authors:** Cheng Hu, Li Wen, Lihui Deng, Chenlong Zhang, Aurelia Lugea, Hsin-Yuan Su, Richard T. Waldron, Stephen J. Pandol, Qing Xia

**Affiliations:** ^1^Department of Integrated Traditional Chinese and Western Medicine, West China Medical School/West China Hospital, Sichuan, China; ^2^Division of Gastroenterology, Department of Medicine, Cedars-Sinai Medical Center, Los Angeles, CA, USA; ^3^Department of Pediatric Gastroenterology, Children's Hospital of Pittsburgh of UPMC and School of Medicine, University of Pittsburgh, Pittsburgh, PA, USA

## Abstract

**Background:**

Environmental factors and genetic mutations have been increasingly recognized as risk factors for chronic pancreatitis (CP). The *PRSS1* p.R122H mutation was the first discovered to affect hereditary CP, with 80% penetrance. We performed here a systematic review and meta-analysis to evaluate the associations of *PRSS1* p.R122H mutation with CP of diverse etiology.

**Methods:**

The PubMed, EMBASE, and MEDLINE database were reviewed. The pooled odds ratio (OR) with 95% confidence intervals was used to evaluate the association of p.R122H mutation with CP. Initial analysis was conducted with all etiologies of CP, followed by a subgroup analysis for hereditary and nonhereditary CP, including alcoholic or idiopathic CP.

**Results:**

A total of eight case-control studies (1733 cases and 2415 controls) were identified and included. Overall, *PRSS1* p.R122H mutation was significantly associated with an increased risk of CP (OR = 4.78[1.13–20.20]). Further analysis showed p.R122H mutation strongly associated with the increased risk of hereditary CP (OR = 65.52[9.09–472.48]) but not with nonhereditary CP, both alcoholic and idiopathic CP.

**Conclusions:**

Our study showing the differential role of p.R122H mutation in various etiologies of CP indicates that this complex disorder is likely influenced by multiple genetic factors as well as environmental factors.

## 1. Introduction

Chronic pancreatitis (CP) is a wide range of progressive fibroinflammatory disease of the exocrine pancreas that eventually leads to irreversible impairment of exocrine and endocrine functions of the gland [1, 2]. The incidence of CP ranges from 4 to 14 per 100,000 worldwide with a higher incidence reported in recent years [3]. Our understanding of the pathogenesis and pathophysiology of CP, including the development of pancreatic fibrosis, etiologic risk factors, natural history, and genetic and epigenetic changes associated, has significantly advanced over time [1, 2, 4, 5]. CP has traditionally been classified as alcohol, hereditary, obstructive, hyperlipidemia, or idiopathic on the basis of etiology [6]. The most common disease phenotype is described as chronic calcifying pancreatitis, which is characterized by clinically apparent acute pancreatitis (AP) at the early stage, progressive development of intraductal stones, pancreatic ductal distortion, strictures, and pancreatic atrophy and extensive destruction of the pancreatic parenchyma leading to steatorrhea and diabetes [3]. Alcohol and smoking are identified as independent risk factors for CP. Both are associated with disease progression, and their risks are likely multiplicative [2]. Several genetic mutations, including *human cationic trypsinogen (PRSS) 1, PRSS2*, *serine protease inhibitor Kazal type 1 gene (SPINK1), chymotrypsinogen C gene (CTRC), transmembrane conductance regulator gene (CFTR), and calcium-sensing receptor (CASR)*, have been noted as risk factors [2, 6]. The role of these gene mutations in CP is becoming increasingly recognized, although the proportion of CP with known genetic mutations is rather small.

*PRSS1* mutation was first discovered to be associated with the phenotype of hereditary pancreatitis (HP) twenty years ago [7]. Carriers with this mutation tend to develop recurrent AP with an early onset of the disease (prior to the second decade of life) and the development of chronic pancreatitis and also have a significantly increased risk for development of pancreatic adenocarcinoma [8, 9]. R122H mutation of the *PRSS1* gene, detected in more than 50% of the patients with HP, was the most common mutation in patients with HP [10]. A single G to A transition mutation in the cationic trypsinogen gene in exon 3 resulted in an arginine (CGC) to histidine (CAC) substitution at amino acid residue 117 of trypsinogen (p.R122H) and this amino acid change eliminates a trypsin cleavage site on the surface of trypsinogen and is predicted to prevent trypsinogen autoinactivation [7, 11]. Mice expressing mouse *PRSS1* mutant p.R122H transgene exhibited early onset of acinar cell injury and inflammation that progressed with age to a chronic stage and associated with fibrosis and acinar cell differentiation [11]. Expression of human *PRSS1* mutants (p.R122H or p.N29I) in murine pancreatic acinar cells also promoted pancreatic injury [12]. Interestingly, endogenously activated trypsinogen (PACE-trypsinogen) in acinar cells caused spontaneous development of AP, but did not exhibit the features of CP, including fibrosis [13]. Genetic deletion of trypsinogen 7, a mouse cationic trypsinogen, caused a 50% reduction in pancreatic damage in caerulein-induced AP [14], but exhibited the same degree of CP induced by repetitive challenges of caerulein compared to wild-type mice [15].

In this study, we performed a systematic review and meta-analysis to investigate the association between p.R122H mutation in *PRSS1* gene and the risk of CP. We used *PRSS1* p.R122H as an example of genetic risk factor in order to understand more about the association of single genetic factor in CP patients. We found that *PRSS1* p.R122H mutation was significantly associated with an increased risk of hereditary CP. Further analysis suggested p.R122H mutation was weakly but still significantly associated with an increased risk of nonhereditary CP, including alcoholic or idiopathic CP with a slightly greater risk in idiopathic CP.

## 2. Methods

### 2.1. Search Strategy

A search of the literature was conducted by two authors (C.H and L.W) independently using PubMed, EMBASE, and MEDLINE electronic databases. The keywords *PRSS1* or p.R122H were combined with *pancreatitis*. The filter “human” was applied. The search was conducted using database entries from January 1996 to July 2016. The language was limited to English. The duplicates were removed manually. We also reviewed reference list of citations in the identified publications for additional citations.

### 2.2. Eligibility Criteria

The published observational studies found by our search that reported the association of chronic pancreatitis with p.R122H mutation were included in this review. Studies that enrolled pediatric patients were excluded (*n* = 36). All basic and animal experimental studies were also excluded (*n* = 26). Review articles (*n* = 67) and case reports (*n* = 39) were excluded. Studies that investigated autoimmune pancreatitis (*n* = 1) or recurrent acute pancreatitis (*n* = 3) were also excluded.

### 2.3. Data Extraction

Data were extracted by two authors (C. H and L. W) independently for each individual study. The following data were extracted: year, study design, racial background, genotyping method, criteria used for diagnosis of CP, number of patients diagnosed as CP, total number of patients, etiology of CP (alcohol, idiopathic, hereditary, and other), history of alcohol consumption, and history of smoking if applicable.

### 2.4. Quality Assessment

The quality of included case-control studies was systematically assessed using the Newcastle Ottawa Scale (NOS). A maximum of 9 points were given to each study within the categories of the selection, comparability, and exposure, as scored by two independent observers. Studies were considered to be “good” quality if they scored >5 points, whereas studies were considered to be “poor” quality if they received ≤5 points.

### 2.5. Definitions

The American Pancreatic Association (APA) guidelines [2] were used to define disease categories. Hereditary CP was defined as present in an individual with CP and with one or two affected first-degree relatives or two or more second-degree relatives affected [16–19]. Nonhereditary CP included idiopathic CP, alcoholic CP, and other etiologies. Idiopathic CP was exclusively diagnosed in the absence of known etiological factors such as alcohol, gallstone, infection, trauma, medications, age over 65, and a positive family history [6].

Alcohol was considered the cause of CP in those who consumed more than 60 or 80 g per day for at least 2 years. Smokers were defined as those who smoked 10 or more cigarettes per day for at least 2 years.

### 2.6. Data Analysis

For each study, the association between *PRSS1* p.R122H mutation and the risk of CP was initially evaluated. A preliminary meta-analysis combining all studies included regardless of etiology was conducted. Further stratified analysis was used to assess the risk of hereditary and nonhereditary CP with *PRSS1* p.R122H mutation, including subanalysis of alcoholic and idiopathic CP. Forest plots were created showing the pooled odds ratio (OR) with the corresponding 95% confidence intervals (CIs). Data were pooled using the random effects model to provide a more conservative estimate. Statistical heterogeneity between studies was determined with Cochran *Q* test and the *I*^2^ value [20]. High statistical heterogeneity was defined as >70%, medium heterogeneity was defined as 50%–70%, and low heterogeneity was defined as 0%–50% [20]. A *p* value of ≤0.05 was considered as statistical significance. Sensitivity analysis was used to test the robustness of associations by omitting each individual study in turn from all available studies when appropriate. Publication bias was assessed by visual inspection of funnel plot [21]. The analysis was performed by Stata software, version SE/13.0 (Stata Corp LP, College Station, Texas, USA).

## 3. Results

### 3.1. Study Selection

**Figure 1:**
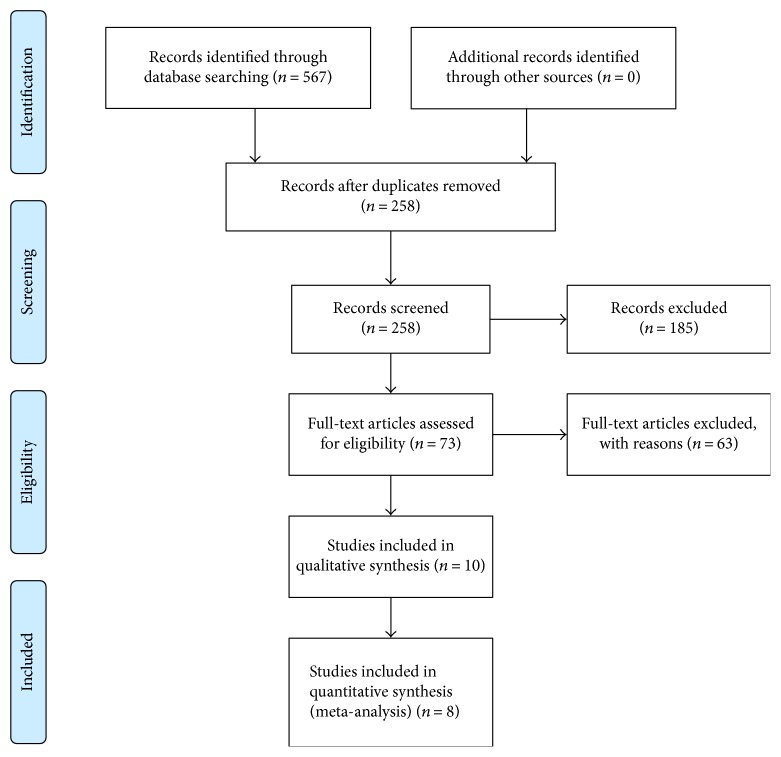
Flow chart of study selection [37].

A total of 567 studies were retrieved from PubMed, EMBASE, and MEDLINE. After removal of duplicates, 258 studies were screened by the title and abstracts. In the preliminary screening, we excluded 185 studies because of the following reasons: pediatric patients (*n* = 36), animal and experimental studies (*n* = 28), case reports (*n* = 39), cross-sectional studies (*n* = 1), review (*n* = 67), editorial (*n* = 2), and others (*n* = 12). After removing, seventy-three articles potentially met the inclusion criteria for eligibility, and 63 were excluded for the reasons as follows: not relevant (*n* = 46), no genotyping for *PRSS1* (*n* = 7), no genotyping for p.R122H (*n* = 6), recurrent AP (*n* = 3), and autoimmune pancreatitis (*n* = 1). Overall, ten studies [16–19, 22–27] were identified and scored for assessing the quality. Two studies were excluded from meta-analysis due to quality score ≤ 5. Finally, eight studies fulfilled completely the criteria for inclusion in this current meta-analysis. The flow diagram showing the study selection process is presented in Figure 1. Methodological quality assessment of the included studies is shown in Supplementary Table 1 available online at https://doi.org/10.1155/2017/9505460.

### 3.2. Study Characteristics

A total of eight case-control-designed studies including CP patients were selected.  Table 1 shows information published for these studies: ethnicity, genotyping methods, and etiologies of CP. Six studies were conducted in Europe and the other two in Asia. First, we analyzed the eight studies that included a total of 1733 cases and 2415 controls. Then, we analyzed the cases based on whether subjects had HP or not (non-HP). Finally, four studies including HP patients (255 cases and 2214 controls) and all eight studies including non-HP patients (1478 cases and 2415 controls) were enrolled. Further analysis of non-HP patients was carried out using six of the studies with ACP patients (308 cases and 575 controls) and seven of the studies with ICP patients (1170 cases and 2315 controls).

### 3.3. Chronic Pancreatitis with All Etiologies Combined

**Figure 2:**
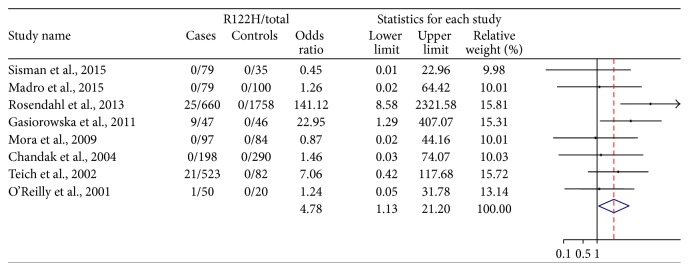
Forest plots showing the association between chronic pancreatitis with all etiologies combined and R122H mutation incidence.

Initial meta-analysis of individual studies and the combined results of the eight studies with all etiologies of CP are shown in Figure 2. The total number of patients from these studies was 1733 CP cases with 2415 healthy controls. The weighted OR revealed an association between p.R122H mutation and that the risk of CP is 4.78 (1.13–20.20). There was low heterogeneity detected between the studies (*p* = 0.171 and *I*^2^ = 32.2%).

### 3.4. Hereditary Chronic Pancreatitis versus Nonhereditary Chronic Pancreatitis

**Figure 3:**
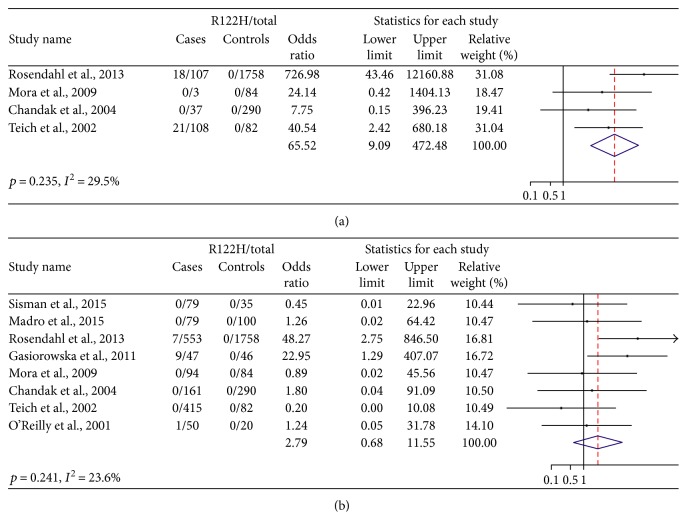
Hereditary CP/R122H mutation versus nonhereditary CP/R122H mutation for case-control studies.

Four of the studies assessed patients with hereditary CP (255 cases and 2214 controls, Figure 3(a)). The p.R122H mutation was detected in 39 of 255 cases and none in 2214 controls. There was low heterogeneity detected between the studies (*p* = 0.235 and *I*^2^ = 29.5%). The pooled OR revealed an association between p.R122H mutation and the risk of hereditary CP of 65.52 (9.09–472.48). Eight studies evaluated patients with nonhereditary CP (1478 cases and 2415 controls, Figure 3(b)). The p.R122H mutation was present in 8 of 1478 cases and none in 2415 controls. There was low heterogeneity detected between the studies (*p* = 0.241 and *I*^2^ = 23.6%). The pooled OR revealed an association between p.R122H mutation, and an increased risk of nonhereditary CP is 2.79 (95%CI, 0.68–11.55). These data indicate that p.R122H mutation is strongly associated with hereditary CP and has a weak association with nonhereditary CP.

### 3.5. Subgroup Analysis of Alcoholic and Idiopathic CP

**Figure 4:**
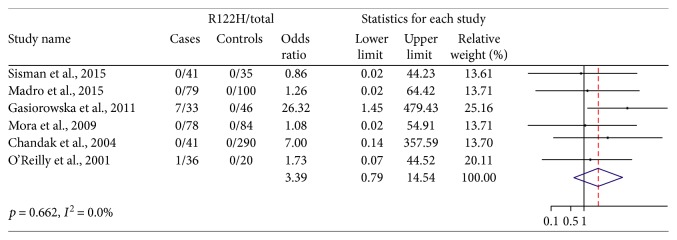
Alcoholic CP/R122H mutation for case-control studies.

Six studies assessed patients with alcoholic CP (308 cases and 575 controls, Figure 4). The p.R122H mutation was detected in 8 of 308 cases and none in 575 controls. There was no heterogeneity detected between the studies (*p* = 0.662 and *I*^2^ = 0.0%). The pooled OR of 3.39 (0.79–14.54) revealed a trend that p.R122H mutation may be associated with an increased risk of alcoholic CP. Since there is only one study with a small size suggesting an association between p.R122H mutation and alcoholic CP and 95%CI crossed 1, we think the p.R122H mutation may not be associated with alcoholic CP, although further study is required to confirm these findings.

**Figure 5:**
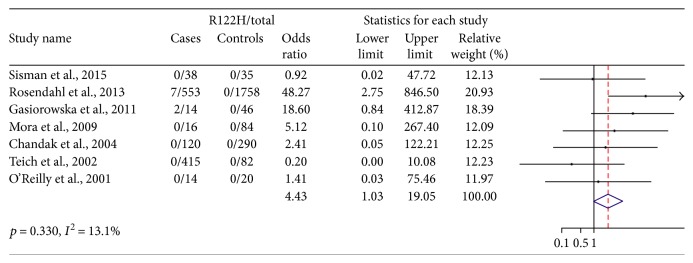
Idiopathic CP/R122H mutation for case-control studies.

Seven studies assessed patients with idiopathic CP (1170 cases and 2315 controls, Figure 5). The p.R122H mutation was detected in 9 of 1170 cases and none in 2315 controls. There was low heterogeneity detected between the studies (*p* = 0.330 and *I*^2^ = 13.1%). The pooled OR is 4.43 (1.03–19.05), which revealed a significant weak association between p.R122H mutation and an increased risk of idiopathic CP. Consistent with the analysis in nonhereditary CP, there was a weak but still significant association between p.R122H mutation and an increased risk of CP.

The sensitivity analysis was performed by sequential omission of each single study in turn. The results indicated that removal of any individual studies one at a time did not change the direction of the pooled effect size (Supplementary Figure 1), indicating that our results are relatively stable and credible.

## 4. Discussion

Overall, we found that the *PRSS1* p.R122H mutation is significantly associated with an increased risk of CP. Our finding is consistent with the meta-analysis conducted by Liu and Zhang [28] which suggested a significant association between total pancreatitis and the *PRSS1* gene. But this study neither specified the sites of the gene mutations nor compared the different pancreatitis etiologies. In our current study, we specifically asked whether there is an association between *PRSS1* p. R122H and chronic pancreatitis with subgroup analysis applied to the known etiologies. Further analysis showed that p.R122H mutation is strongly associated with hereditary CP. Since the first report in 1996 indicating that *PRSS1* p.R122H mutation was associated with the phenotype of HP in a North American family [7], strong associations between the p.R122H mutation in the *PRSS1* gene and HP have been reported in a number of studies from many parts of the world including Europe and Asia [29–31]. Subgroup analysis for the known etiologies, including alcoholic and idiopathic CP, showed that p.R122H mutation is weakly correlated with an increased risk of nonhereditary CP. In recent years, smoking has been recognized as an independent risk factor affecting the course and progression of CP [32, 33]. We were unable to investigate the association of smoking with the risk of CP as there is lack of reported data extracted from the studies we included in our analysis.

Various *PRSS1* gene mutations in exon 2 and exon 3 have been reported to be pathogenic and associated with the phenotype of CP. p.R122H and p.N29I mutations are the most common mutations [34]. Recently, the genome-wide association study (GWAS) in alcoholic CP discovered that the common genetic variants in the *PRSS1-PRSS2* locus account for an increased risk for alcoholic and sporadic CP, but rare *PRSS1* gene variants, including three known diseases that were also screened and identified, were not associated with the observed phenotype [35]. This finding was replicated and refined in a large European cohort by an independent group [36]. Both studies suggested the importance of continuing to identify disease-associated genetic variants.

In summary, our findings indicate that the *PRSS1* p.R122H mutation is significantly associated with an increased risk of CP overall. The differential role of *PRSS1* p.R122H mutation was observed in both hereditary and nonhereditary CP. These data suggest that multiple genetic and environmental factors determine the initiation and progression of CP.

## Supplementary Material

Supplementary Table 1 Methodologic Quality assessment of included studies according to the Newcastle-Ottawa Scale. Supplementary Figure 1 Sensitivity analysis on the studies.

## Figures and Tables

**Table 1:** Characteristics of case-control studies included.

Author, year	Journal	Genotyping method	Author country	Study population	Non-HP		HP
					ACP	ICP	
Sisman et al., 2015 [27]	Turk J Gastroenterol	RFLP-PCR	Turkey	Turkey	+	+	
Madro et al., 2015 [26]	Gastroenterol Res Pract	RT-PCR	Poland	N/A	+		
Rosendahl et al., 2013 [16]	Gut	PCR	Germany	German		+	+
Gasiorowska et al., 2011 [25]	Dig Dis Sci	RFLP-PCR	Poland	Polish	+	+	
Mora et al., 2009 [17]	Pancreatology	RFLP-PCR	Spain	Spanish	+	+	+
Chandak 2004 [18]	Gut	—	India	Indian	+	+	+
Teich et al., 2002 [19]	Am J Gastroenterol	PCR	Germany	N/A		+	+
O'Reilly et al., 2001 [22]	Digestion	PCR	UK	Ethnically matched	+	+	

N/A: not available.
