# Supplementary material for: The Differential Role of Human Cationic Trypsinogen (PRSS1) p.R122H Mutation in Hereditary and Nonhereditary Chronic Pancreatitis: A Systematic Review and Meta-Analysis
Source: Gastroenterol Res Pract. 2017 Oct 8;2017:9505460. doi: 10.1155/2017/9505460 (PMC5651130; doi:10.1155/2017/9505460)
Supplement: Supplementary file 1 — Supplementary Table 1 Methodologic Quality assessment of included studies according to the Newcastle-Ottawa Scale. Supplementary Figure 1 Sensitivity analysis on the studies. [file 9505460.f1.docx]

| Study | Selection | Comparability | Exposure | Total |
| --- | --- | --- | --- | --- |
| O'Reilly et al., 2001 | 2 | 1 | 3 | 6 |
| Teich et al., 2002 | 2 | 1 | 3 | 6 |
| Chandak et al., 2004 | 3 | 1 | 3 | 7 |
| Keiles et al., 2006 | 2 | 1 | 2 | 5 |
| LlU et al., 2008 | 1 | 1 | 2 | 4 |
| Mora et al., 2009 | 3 | 1 | 3 | 7 |
| Gasiorowska et al., 2011 | 2 | 1 | 3 | 6 |
| Rosendahl et al., 2013 | 3 | 1 | 3 | 7 |
| Madro et al., 2015 | 4 | 1 | 3 | 8 |
| Sisman et al., 2015 | 2 | 1 | 3 | 6 |

**Supplementary Table 1 Methodologic Quality assessment of included studies according to the Newcastle-Ottawa Scale**
