# Supplementary material for: The Differential Role of Human Cationic Trypsinogen (PRSS1) p.R122H Mutation in Hereditary and Nonhereditary Chronic Pancreatitis: A Systematic Review and Meta-Analysis
Source: Gastroenterol Res Pract. 2017 Oct 8;2017:9505460. doi: 10.1155/2017/9505460 (PMC5651130; doi:10.1155/2017/9505460)

**Supplementary Figure1 Sensitivity analysis on the studies**

**A sensitivity analysis on chronic pancreatitis with all etiologies combined studies**


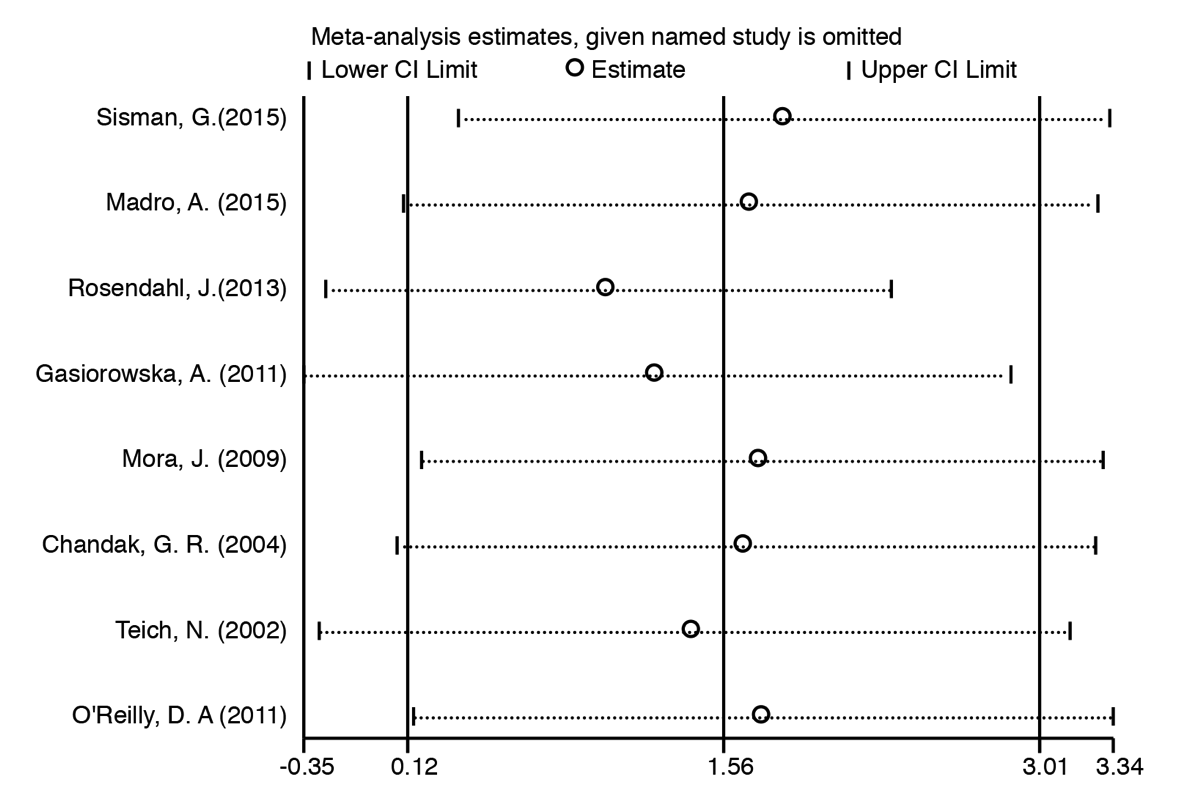


**B sensitivity analysis on Hereditary CP studies**


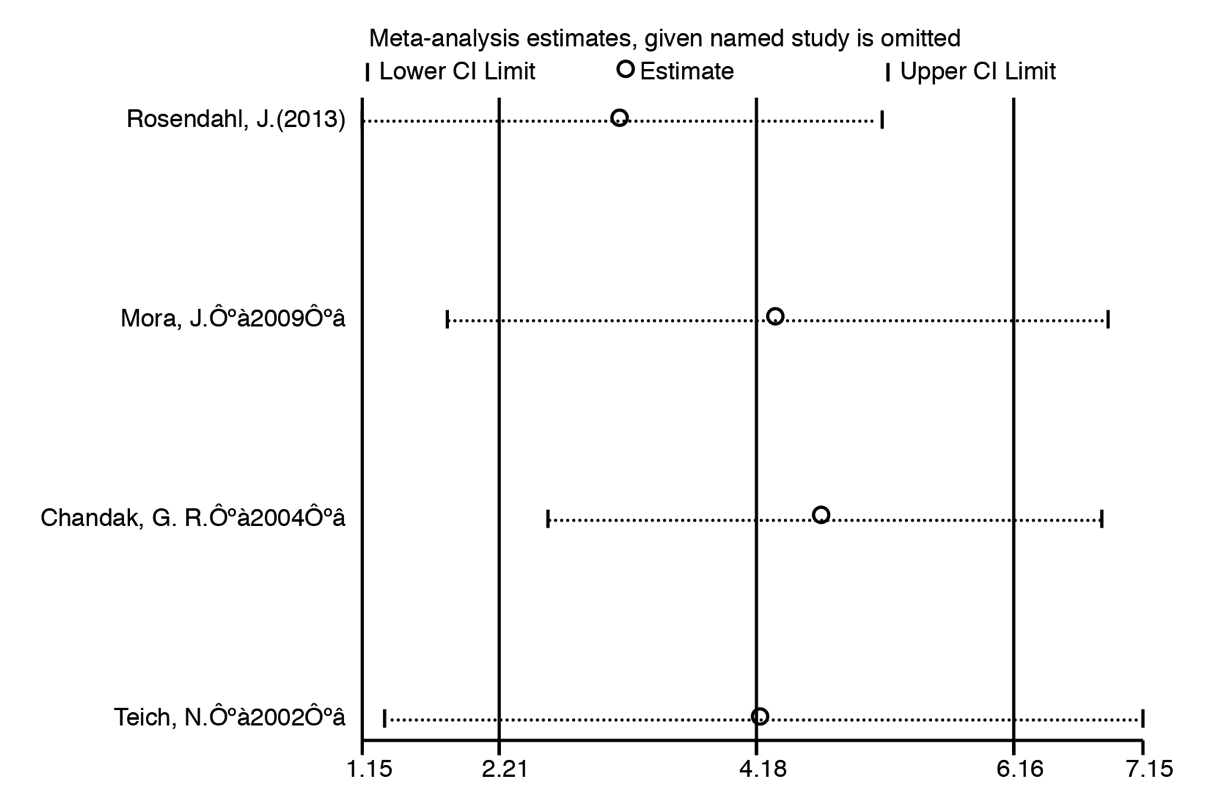


‘

**C sensitivity analysis on non-Hereditary CP studies**


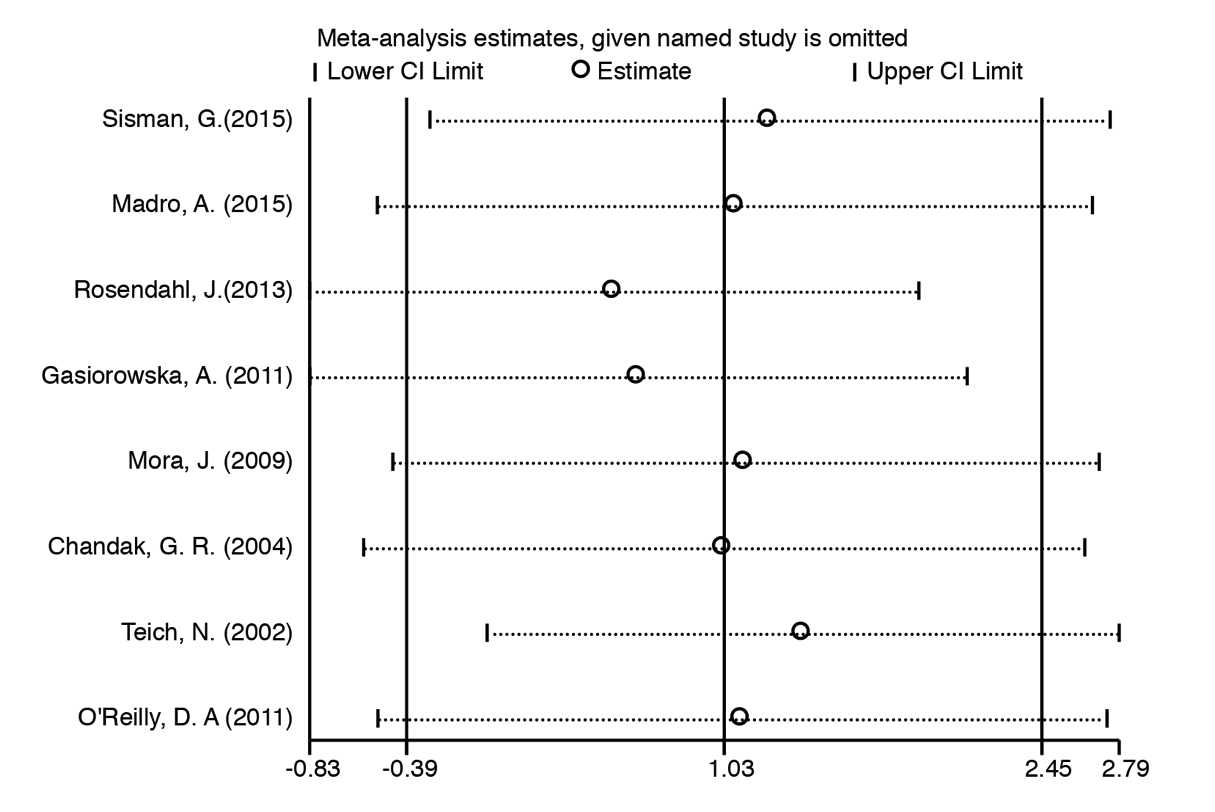

Supplement: Supplementary file 2 [file 9505460.f2.docx]
